# Marine and freshwater regime changes impact a community of migratory Pacific salmonids in decline

**DOI:** 10.1111/gcb.15895

**Published:** 2021-10-20

**Authors:** Kyle L. Wilson, Colin J. Bailey, Trevor D. Davies, Jonathan W. Moore

**Affiliations:** ^1^ Earth to Oceans Research Group Department of Biological Sciences Simon Fraser University Burnaby British Columbia Canada; ^2^ Central Coast Indigenous Resource Alliance Campbell River British Columbia Canada; ^3^ B.C. Ministry of Forests, Lands and Natural Resource Operations and Rural Development, Fish and Aquatic Habitat Branch Victoria British Columbia Canada

**Keywords:** Bayesian, ecosystem change, fisheries, population dynamics, salmon, time‐series, watersheds

## Abstract

Marine and freshwater ecosystems are increasingly at risk of large and cascading changes from multiple human activities (termed “regime shifts”), which can impact population productivity, resilience, and ecosystem structure. Pacific salmon exhibit persistent and large fluctuations in their population dynamics driven by combinations of intrinsic (e.g., density dependence) and extrinsic factors (e.g., ecosystem changes, species interactions). In recent years, many Pacific salmon have declined due to regime shifts but clear understanding of the processes driving these changes remains elusive. Here, we unpacked the role of density dependence, ecosystem trends, and stochasticity on productivity regimes for a community of five anadromous Pacific salmonids (Steelhead, Coho Salmon, Pink Salmon, Dolly Varden, and Coastal Cutthroat Trout) across a rich 40‐year time‐series. We used a Bayesian multivariate state‐space model to examine whether productivity shifts had similarly occurred across the community and explored marine or freshwater changes associated with those shifts. Overall, we identified three productivity regimes: an early regime (1976–1990), a compensatory regime (1991–2009), and a declining regime (since 2010) where large declines were observed for Steelhead, Dolly Varden, and Cutthroat Trout, intermediate declines in Coho and no change in Pink Salmon. These regime changes were associated with multiple cumulative effects across the salmon life cycle. For example, increased seal densities and ocean competition were associated with lower adult marine survival in Steelhead. Watershed logging also intensified over the past 40 years and was associated with (all else equal) ≥97% declines in freshwater productivity for Steelhead, Cutthroat, and Coho. For Steelhead, marine and freshwater dynamics played approximately equal roles in explaining trends in total productivity. Collectively, these changing environments limited juvenile production and lowered future adult returns. These results reveal how changes in freshwater and marine environments can jointly shape population dynamics among ecological communities, like Pacific salmon, with cascading consequences to their resilience.

## Graphical abstract

We found that recent declines in a community of migratory Pacific salmon were linked to 40 years of marine and freshwater regime changes. These stressful changes included the following: increased seal abundances, increased competition in the ocean, oceanic oscillations, warming climates, and increased watershed logging. Each stressor had unique impacts at different points in salmon life cycle, such as reduced marine survival of returning adults, altered spawning migration timing, or reduced freshwater survival in early life. Cumulatively, these stressors are keeping many salmon populations bottlenecked at critically low abundances.

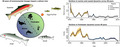

## 1 INTRODUCTION

Ecosystems are increasingly challenged by the cumulative impacts from anthropogenic stressors that can induce unexpected shifts in ecological regimes (Möllmann et al., 2015; Rocha et al., 2018; Scheffer & Carpenter, 2003). Regime shifts are large, rapid, and persistent changes in ecosystem processes that can have major impacts on ecosystem services and are increasingly observed in aquatic (e.g., lake eutrophication, marine overfishing), coastal (e.g., salt marsh conversion to tidal flats), terrestrial (e.g., forest‐to‐savanna conversion), and global ecosystems (e.g., Arctic ice caps; Rocha et al., 2018). The impacts from these regime shifts can cascade within and across ecological scales like the ‘sequential collapse of a line of dominoes’ (Carpenter & Brock, 2004). For example, while regime shifts were often historically characterized as two alternating states (Biggs et al., 2018), recent studies demonstrate that regime shifts may trigger subsequent regime shifts that challenges current management and conservation paradigms (Francis et al., 2021; Hempson et al., 2018).

The combined impacts of slow and fast environmental changes, such as gradual selective forcing or environmental fluctuations, can drive persistent (i.e., nonstationary) regime shifts in productivity (Litzow et al., 2018; Shelton & Mangel, 2011; Vert‐pre et al., 2013). Density dependence can structure stationary regimes of population regulation that allows populations to buffer themselves against some environmental changes (Brännström & Sumpter, 2005; Shelton & Mangel, 2011). For example, populations often compensate for decreased abundances with increased productivity or survival (i.e., compensatory capacity) typically from reduced competition for otherwise limited resources (Brännström & Sumpter, 2005). However, the compensatory capacity of populations to cope with change may be limited by their environment or life histories (Allendorf et al., 2008; Shelton & Mangel, 2011). This constrained compensation may leave populations vulnerable to pressures from environmental changes that may induce nonstationary population dynamics (Hempson et al., 2018). For example, climatic changes can drive populations into periods of lower productivity or survival, weakening the natural feedbacks that previously maintained and recovered populations (Biggs et al., 2018; Litzow et al., 2018).

Marine regime shifts have been described several times in Pacific salmonids (e.g., Mantua & Hare, 2002)—a group of migratory anadromous fishes that provide important function and services to marine, coastal, and freshwater ecosystems (Schindler et al., 2003). Salmonid populations are structured by marine and freshwater environments and can exhibit wide fluctuations in productivity, survival, and population dynamics (Mantua & Hare, 2002; Rogers et al., 2013). These fluctuations emerge from and shape a complex interplay between intrinsic and extrinsic factors, like density dependence, environmental variation, or species interactions (Schindler et al., 2008). For example, recent trends in marine and oceanographic indices, like the North Pacific Gyre Oscillation, have been linked with persistent changes in recruitment productivity regimes and lower production of wild Pacific salmonids (Dorner et al., 2018; Malick et al., 2017; Mueter et al., 2002), but these relationships appear to be weakening (Kilduff et al., 2015). These regime shifts alter fishery quotas and recovery timelines and are associated with the widespread decline of wild Pacific salmonids across the Pacific Northwest (Dorner et al., 2013; Peterman & Dorner, 2012; Teresa A’Mar et al., 2009). Despite marine regime shifts having been frequently observed in Pacific salmonids (Welch et al., 2020), descriptions of freshwater regimes shifts are rare (but see Atlas et al., 2015; Jones et al., 2020; Scheuerell et al., 2020), and relatively few studies have assessed regime shifts in both marine and freshwater contexts. Moreover, studies generally focus on a single species even though salmon rivers often support diverse communities.

**FIGURE 1:**
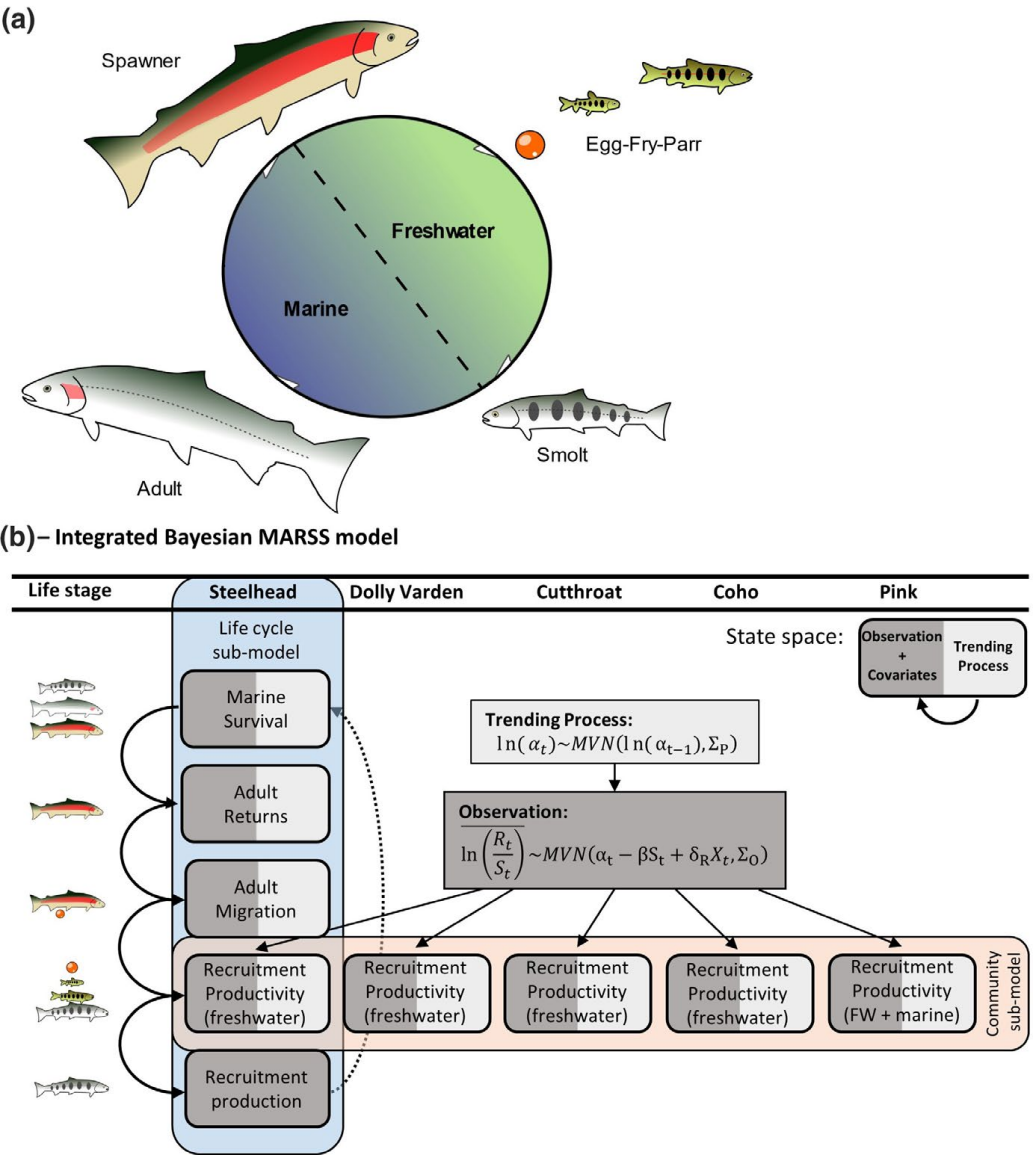
(a) Anadromous salmonid life cycle and population dynamics depend on freshwater and marine environments that can carry over across generations. (b) Conceptual overview for integrated Bayesian multivariate autoregressive state‐space model evaluating Keogh population dynamics

Here, we estimated the relative roles of density dependence, ecosystem trends, and stochasticity on recruitment productivity regimes for a salmonid community across a rich 40‐year time‐series using an integrated Bayesian multivariate autoregressive state‐space (MARSS) model (Figure 1). Our objective was to quantify the intrinsic and extrinsic factors associated with changing productivity within and across the life cycles of a diverse salmonid community that experiences similar marine and freshwater environments. Overall, this study reveals how cumulative effects from both marine and freshwater environments can jointly shape and bottleneck productivity regimes for Pacific salmonid communities.

## 2 METHODS

### 2.1 Study site

**FIGURE 2:**
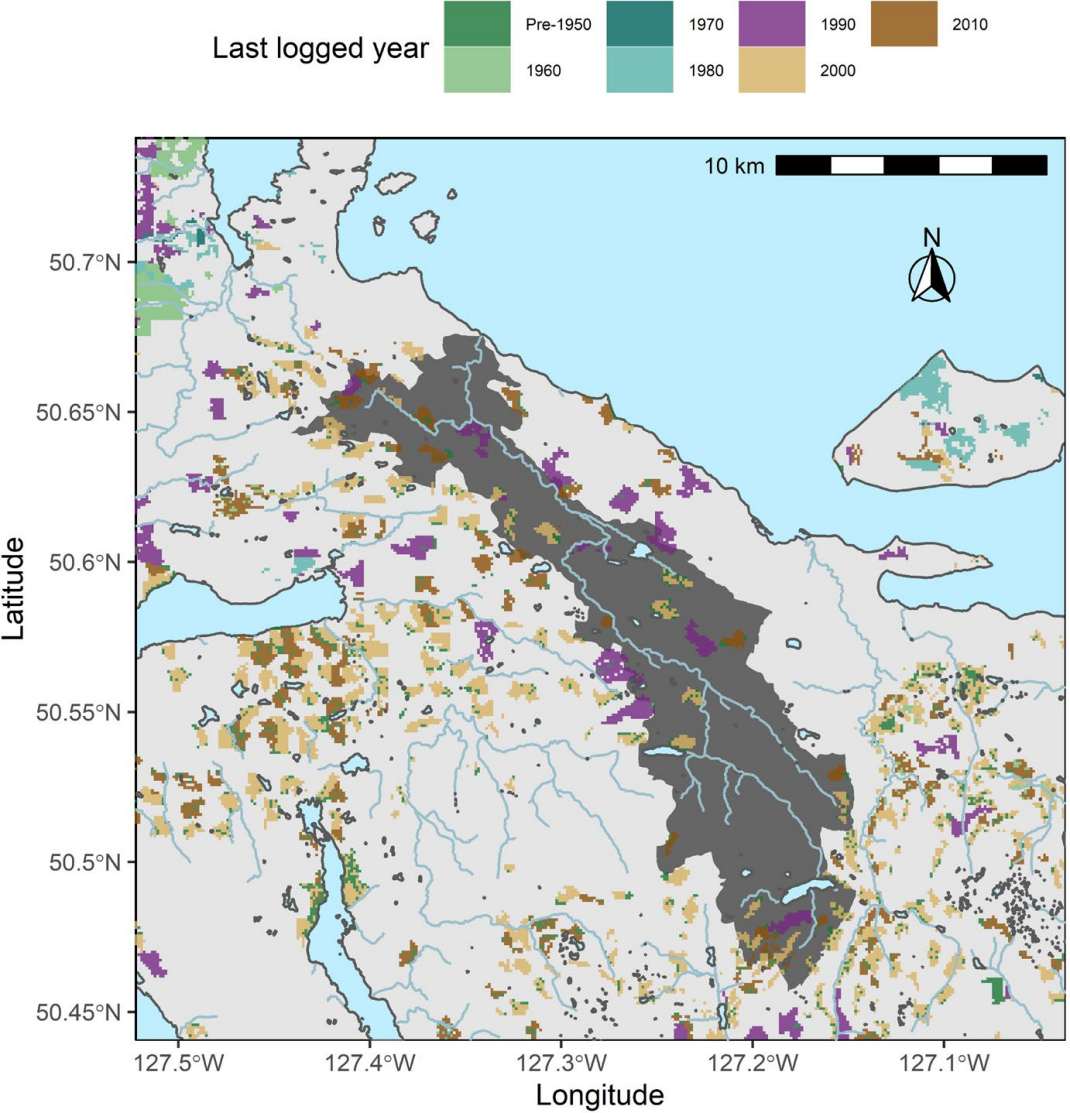
Keogh River watershed (dark region) and historical logging activity (coloured polygons)

The Keogh River (Giyuxw—river name from the local Kwakiutl First Nation) is a small (31.2 km long, 130 km^2^ watershed area), lake‐headed, and rain‐dominated coastal watershed in northern Vancouver Island, British Columbia (Figure 2). The Keogh River currently supports five species of anadromous Pacific salmonids: Coho Salmon (*Oncorhynchus kisutch*), Pink Salmon (*O*. *gorbuscha*), Steelhead Trout (*O*. *mykiss*), Coastal Cutthroat Trout (*O*. *clarkii clarkii*), and Dolly Varden (*Salvelinus malma*), each of which varies in their life histories (Table 1; Figure 1a). Each species’ life cycle can generally be defined as a cohort of individuals that (a) were deposited as eggs into the gravel in their respective brood‐year from a cohort of reproducing adults and hatched the following spring, (b) out‐migrated from the river to the coastal Pacific Ocean as smolts some years later (age varies by species), and (c) returned to the Keogh River for reproduction after some years in the North Pacific following their smolt out‐migration (age varies by species, cohort, and individuals). The abundance of adult and juvenile Pacific salmonids was measured annually since 1976 using a combination of methods: (1) a counting fence and trap that spans the full river during important windows for upstream and downstream migrations, (2) mark‐recapture studies of returning adult Steelhead, (3) a resistivity counter installed in 1997, and (4) adult abundance estimates from stream bank walks pre‐1997 (Fisheries & Oceans Canada, 2020). For adult Coastal Cutthroat and Dolly Varden, we relied on counts of adults migrating back downstream after spawning where spawning mortality would downwardly bias adult abundances for a given brood‐year. We thus assumed post‐spawn adult counts were proportional to total spawners such that our measure for population productivity for these two species would be relatively precise year‐to‐year. Our compiled time‐series of 1976–2015 covered all cohorts that completed a life cycle (i.e., freshwater rearing, migration, and spawning) since sampling began. More detailed information on the Keogh River and its research history can be found in Bailey et al., (2018).

**TABLE 1:** Species life cycles defined by their life stages (with brood‐year and spawn‐year ***t***), capture method, lifespan (years), and the mean age‐ and size‐structure (mean fork length; mm) during the freshwater and marine portion of their life cycle

Species	Life stages		Lifespan (yrs)	Freshwater		Marine	
	Recruit	Spawner		Age	FL (mm)	Age	FL (mm)
Dolly Varden	Smolt (t)^1^	Adult (t)^2^	4.7 (1–9)	1.7 (0–4)	150	3 (1–4)	261
Steelhead	Smolt (t)^1^	Adult (t)^3−5^	5.3 (2–9)	2.3 (0–4)	176	2.5 (1–5)	721
Coastal cutthroat	Smolt (t)^1^	Adult (t)^2^	5.9 (4–8)	3 (2–4)	157	2.9 (2–4)	230
Coho salmon	Smolt (t)^1^	Adult (t)^5,6^	3	1	103	2	660
Pink salmon	Adult (t+2)^5,6^	Adult (t)^5,6^	2	0	30	2	472

Age‐structure for Dolly Varden and Coastal Cutthroat based on prior work on the Keogh River (Smith & Slaney, 1980).

Capture and enumeration methods include the following: ^1^Downstream Fish Fence (out‐migration); ^2^Downstream Fish Fence (post‐spawn adults); ^3^Upstream Fish Fence; ^4^Angling Mark Recapture; ^5^Resistivity Counter 1997‐present; ^6^Stream Walks (before 1997).

### 2.2 Marine and freshwater environment

We used 11 indices compiled from 1976 to characterize the marine and freshwater environmental factors hypothesized to affect each of the five examined species (Atlas et al., 2015; Bailey et al., 2018). The freshwater environment was characterized using eight indices to describe the following: inland climate—measured as (1) mean winter (November–February) air temperatures, (2) mean summer (March–September) air temperatures, (3) total winter rainfall, and (4) total summer rainfall from climate data taken near the mouth of the Keogh River at the Port Hardy airport (LaZerte & Albers, 2018); forestry impacts—measured using (5) the 15‐year cumulative area of logging activity (km^2^) in the watershed (Bourgeois et al., 2018); phenology—measured as (6) the median date of the adult migration in a cohorts’ spawning years (only available for Steelhead Trout); nutrient enrichment—measured as (7) the occurrence of whole‐river inorganic nutrient addition treatments applied from 1983 through 1986 and from 1997 through 2004; and facilitative species interactions—measured as (8) the abundance of Pink Salmon spawning in a cohorts’ brood‐year. We estimated uncertainty in median date of spawning run and the date of the fence count installation in the pre‐1997 arrival dates using data available from when the resistivity counter that was installed post‐1997 to sample adult spawners year‐round (Figure S1).

The marine environment was characterized using three indices describing: coastal predation—measured as (9) coastal densities of harbour seals in the Strait of Georgia (Nelson et al., 2019) as an index for the relative abundance of marine predators; marine competition—measured as (10) the North Pacific salmon abundance (NPSA; Ruggerone & Irvine, 2018); and marine climate—measured as (11) the North Pacific Gyre Oscillation index (NPGO; Malick et al., 2017). As seal densities and NPSA were both collinear, we combined the two indices using the first two component axes from a principal component analysis. We termed the first PCA axis as “ocean interactions” describing a combined index of predation and competition in the marine portion of each species’ life cycle, and the second principal components axis as “ocean PCA‐2,” which was positively associated with seal densities and negatively associated with NPSA. All remaining marine and freshwater covariates used for our analyses had variance inflation factors ≤4.0 (with 35 of 39 species‐specific covariates ≤2.0) suggesting multicollinearity among predictors was not problematic (Zuur et al., 2009).

Annual environmental data were matched to brood‐years using the weighted average of observed species age‐structure and appropriate time‐lags (Table 1). For example, the freshwater conditions of the 1980 steelhead smolt cohort was the weighted average of the 1975–1979 freshwater environment using the proportions‐at‐age of each cohort. Our index of seal densities were measured as the summed seal densities from a cohort's out‐migrating year and their subsequent spawning year, assuming smolts and migrating adults can both be subjected to seal predation (except for juvenile Pink Salmon, whose small smolt body sizes are thought to be below the seal predation window, and thus, we only considered potential seal predation on adult Pink Salmon; Thomas et al., 2017). We assumed a 15‐year lag time for the cumulative impact of forestry—other studies suggest forestry lag‐times of 10–30 years (Gronsdahl et al., 2019; Tschaplinski et al., 2007; Tschaplinski & Pike, 2017). We ran sensitivity tests on the forestry lag time, ranging from 5 to 30 years, and found relatively consistent inferences in species’ responses to cumulative logging (Figure S2). Although estimates of logged area on Vancouver Island may be conservative due to inconsistent reporting, our measurements closely tracked estimates of land‐use disturbance in the Keogh watershed reconstructed from remote sensing and satellite imagery (Shackelford et al., 2018). Missing years of annual adult (21 of 200 adult cohorts were missing, ranging from 0 to 9 across species), juvenile (3 of 200 juvenile cohorts were missing, all Dolly Varden), and environmental data (<1% of covariate‐year combinations were missing, ranging from 0 to 4 across covariates) from 1976 to 2018 were reconstructed using a dynamic factor analysis in the “MARSS” package in R version 4.0.2 (Holmes et al., 2012, 2020; R Core Team, 2021) that allowed for covariation between datasets to impute maximum likelihood estimates for missing years (Figures S2 and S3).

### 2.3 Integrated model

We developed an integrated Bayesian MARSS model to describe nonstationary trends in recruitment productivity for the Keogh River salmonid community across 40 years (Figure 1b). Multivariate state‐space models are a useful approach to analyse time‐series data because they can simultaneously estimate trends in explanatory covariates of the time‐series while accounting for the temporal dependency between observations (i.e., the autoregressive property), correlations between two or more time‐series (i.e., the multivariate property), and two sources of uncertainty: observation error and natural biological variation (i.e., the observation vs. process states). We developed our MARSS model to estimate trends in recruitment productivity—defined as the number of recruits (i.e., offspring in subsequent generation) produced per spawning adult of a given year (called a ‘brood‐year’). Our model integrated two core analyses: (1) a whole‐community analysis of trends in recruitment productivity, species covariation, and environmental associations and (2) a within‐species analysis of the trends, linkages, and associations between marine survival, adult returns, adult upstream migration timing, smolt recruitment productivity, and smolt recruitment for Steelhead Trout. The observed recruitment of four species (Dolly Varden, Steelhead Trout, Coastal Cutthroat, and Coho Salmon) was defined as the abundance of smolt cohorts out‐migrating from freshwater rearing sampled at the smolt fence. However, the observed recruitment for Pink Salmon was defined as the abundance of returning adults 2 years after their brood‐year. Hence, we included only freshwater environmental covariates for species with smolt monitoring, while Pink Salmon recruitment included both freshwater and marine covariates.

The first submodel assessed potential trends and associations on recruitment productivity (predominately freshwater), which was defined using a Ricker model (Brännström & Sumpter, 2005; Ricker, 1975) that describes a nontrending (i.e., stationary) density‐dependent relationship between adult stock *S* and recruitment *R*. The basic Ricker stock–recruitment model follows:

(1) $$R_{t}=\alpha S_{t}e^{‐\beta S_{t}}$$

where α is the number of recruits produced per unit spawner at low densities (i.e., maximum intrinsic productivity) and β reflects the strength of density dependence such that $\frac{1}{\beta }$ is the adult abundance that produces the maximum number of recruits. We linearized the Ricker model into a simpler regression‐like form such that:

(2) $$\ln\left(\frac{R_{t}}{S_{t}}\right)=\ln\left(\alpha \right)‐\beta S_{t}$$

where $\ln\left(\frac{R_{t}}{S_{t}}\right)$ measures the observed recruitment productivity in year *t*. We then added environmental covariates ${X_{R}}_{t}$ into this form such that:

(3) $$\ln\left(\frac{R_{t}}{S_{t}}\right)=\ln\left(\alpha \right)‐\beta S_{t}+\delta {X_{R}}_{t}$$

Hence, a linearized Ricker model can be turned into a multiple linear regression to address how intrinsic productivity (*α*), density‐dependence (*β*), and environmental drivers ${X_{R}}_{t}$ jointly shape recruitment productivity in Pacific salmonids. However, Equation (3) assumes that the stock–recruitment relationship, defined by *α* or *β*, remains constant through time, which leads to a stationary productivity regime that responds to changing adult densities or environmental conditions.

Consistent with a MARSS model, we then added a time‐varying component (i.e., nonstationary) to Equation (3) to allow us to estimate persistent and systematic trends in productivity regimes. Time‐varying dynamics were modelled as $\ln\left(\alpha_{t}\right)$ or $\beta_{t}$ that varies at time *t* such that:

(4) $$\ln\left(\frac{R_{t}}{S_{t}}\right)=\ln\left(\alpha_{t}\right)‐\beta_{t}S_{t}+\delta {X_{R}}_{t}+v_{t}$$

(5) $$\ln\left(\alpha_{t}\right)=\ln\left(\alpha_{t‐1}\right)+u_{t}$$

where Equation (4) is the observation state with error $v_{t}$ and Equation (5) is the process state showing that annual productivity *α* follows an autoregressive (lag 1) process with normally distributed error $u_{t}$ with mean zero and standard deviation σ (and the same process state would follow for a time‐varying $\beta_{t}$). Modelling recruitment as a time‐varying process allowed us to estimate and detect trends in productivity regimes (Dorner et al., 2008). If there were no evidence for a trend, then estimates for $u_{t}$ in the process state would drop towards zero leading to a constant and stationary regime. Preliminary results using approximate leave‐one‐out cross validation in the package “loo” (Vehtari et al., 2017) for model selection strongly favored evidence of shifting $\ln\left(\alpha_{t}\right)$ rather than shifting $\beta_{t}$ (or both) in the linearized Ricker model, similar to Dorner et al., (2008). Our analysis for trends in recruitment productivity regimes was thus evaluated in two ways: (a) observed recruitment productivity—the observed recruits produced per adult spawner $\ln\left(\frac{R_{t}}{S_{t}}\right)$ explained as a function of changing adult densities and environmental drivers, and (b) estimated intrinsic productivity—the nonstationary shifts in the maximum intrinsic productivity $\ln\left(\alpha_{t}\right)$, i.e., a time‐varying intercept of the stock–recruitment relationship that is the predicted number of recruits per spawner at low population abundance.

We then allowed for annual variation in observed recruitment productivity and intrinsic productivity $\ln\left(\alpha_{t}\right)$ to (co)vary among species using a multivariate distribution. Specifically, we characterized population productivity using a multivariate normal distribution with a mean vector $\bar{\ln\left(\frac{R_{t}}{S_{t}}\right)}$ describing the expected annual productivity of five species at time *t* and a variance‐covariance matrix $\Sigma_{O}$ modelling observation error within and between species productivity. Our observation model, thus, followed:

(6) $$\bar{\ln\left(\frac{R_{t}}{S_{t}}\right)}=\left(\begin{array}{c} \ln\left(\frac{R_{t,1}}{S_{t,1}}\right)\\ \ln\left(\frac{R_{t,2}}{S_{t,2}}\right)\\ \ln\left(\frac{R_{t,3}}{S_{t,3}}\right)\\ \ln\left(\frac{R_{t,4}}{S_{t,4}}\right)\\ \ln\left(\frac{R_{t,5}}{S_{t,5}}\right) \end{array}\right)=\left(\begin{array}{c} \begin{array}{c} \begin{array}{c} \begin{array}{c} \ln\left(\alpha_{t,1}\right)‐\beta_{1}S_{t,1}+\delta_{1}x_{t,1}\\ \ln\left(\alpha_{t,2}\right)‐\beta_{2}S_{t,2}+\delta_{2}x_{t,2} \end{array}\\ \ln\left(\alpha_{t,3}\right)‐\beta_{3}S_{t,3}+\delta_{3}x_{t,3} \end{array}\\ \ln\left(\alpha_{t,4}\right)‐\beta_{4}S_{t,4}+\delta_{4}x_{t,4} \end{array}\\ \ln\left(\alpha_{t,5}\right)‐\beta_{5}S_{t,5}+\delta_{5}x_{t,5} \end{array}\right)∼\text{MVN}\left(\alpha_{t}‐\beta S_{t}+\delta_{R}X_{Rt^{\prime }}\Sigma_{O}\right)$$

where $\ln(\alpha_{t})$ was the autoregressive time‐varying process state such that:

(7) $$\ln\left(\alpha_{t}\right)=\left(\begin{array}{c} \begin{array}{c} \begin{array}{c} \begin{array}{c} \ln(\alpha_{t,1})\\ \ln(\alpha_{t,2}) \end{array}\\ \ln(\alpha_{t,3}) \end{array}\\ \ln(\alpha_{t,4}) \end{array}\\ \ln(\alpha_{t,5}) \end{array}\right)=\left(\begin{array}{c} \begin{array}{c} \begin{array}{c} \begin{array}{c} \ln(\alpha_{t‐1,1})\\ \ln(\alpha_{t‐1,2}) \end{array}\\ \ln(\alpha_{t‐1,3}) \end{array}\\ \ln(\alpha_{t‐1,4}) \end{array}\\ \ln(\alpha_{t‐1,5}) \end{array}\right)∼\text{MVN}\left(\ln\left(\alpha_{t‐1}\right),\Sigma_{P}\right)$$

We used the Cholesky decomposition to estimate the variance‐covariance matrices ($\Sigma_{O}$ and $\Sigma_{P}$, each a 5 × 5 matrix) as the product of a lower‐triangular correlation matrix and a diagonal element of five within‐species variances that were each positive‐constrained and log‐transformed (Stan Development Team, 2021).

### 2.4 Steelhead life cycle

Our second submodel integrated major aspects of the marine and freshwater portions of the Steelhead Trout life cycle to evaluate how marine or freshwater regime shifts may affect a species. Specifically, we linked Steelhead smolt out‐migration, marine survival, adult returns, spawning migration timing, and recruitment productivity.

We modelled the marine survival $m_{t}$ of smolts out‐migrating from freshwater environment as a function of marine and coastal drivers following a logit regression:

(8) $$\text{logit}\left(m_{t}\right)=m_{0_{t}}+\delta_{m}X_{m_{t}}$$

where $\delta_{m}$ were the coefficients for environmental covariates $X_{m_{t}}$ and average marine survival was allowed to trend through time following:

(9) $$m_{0_{t}}=m_{0_{t‐1}}+u_{m_{t}}$$

As monitoring on the Keogh River began in 1976, we estimated the observed logit‐transformed marine survival of the first spawner cohort in the time‐series as incomplete (missing) data imputed from a vague normal prior.

We then modelled the number of returning adult female Steelhead $S_{t}$ to the Keogh River as a function of logit‐marine survival (eq. 8 above) and the cohort abundance of their out‐migration from freshwater $F_{t}$ following:

(10) $$S_{t}={S_{0}}_{t}+\delta_{S_{1}}m_{t}+\delta_{S_{2}}F_{t}$$

where $\delta_{S_{1}}$ and $\delta_{S_{2}}$ were the effect of marine survival and freshwater cohorts, respectively, on adult returns, and the intercept for adult females ${S_{0}}_{t}$ was allowed to trend through time following:

(11) $$S_{0_{t}}=S_{0_{t‐1}}+u_{S_{t}}$$

Next, we modelled the median date of the upstream adult migration as a function of both coastal and freshwater drivers (mean air temperature and total rainfall 14 days before upstream migration) and spawner abundance (assuming a log‐linear density‐dependent relationship) from Equation (10) following:

(12) $$T_{t}={T_{0}}_{t}+\delta_{T_{S}}{\ln(S}_{t})+\delta_{T}{X_{T}}_{t}$$

where $\delta_{T}$ were the coefficients for environmental drivers ${X_{T}}_{t}$ on the timing of the upstream adult migration, and the average date of the adult migration was allowed to trend through time following:

(13) $${T_{0}}_{t}={T_{0}}_{t‐1}+u_{T_{t}}$$

We closed the Steelhead life cycle by using eq. 6 to model freshwater production of smolts as a function of returning adults (abundance and timing), temperature and rainfall during staging, nesting, or egg incubation (measured as mean temperature and total rainfall 30 days after median upstream adult migration), and other annual‐level freshwater environments thought to affect their freshwater rearing (i.e., logging within the watershed) following Equation (6). Integrating Equations ((8), (9), (10), (11), (12), (13)) with Equations (6) and (7) formally linked the marine and freshwater life cycles of Steelhead Trout on the Keogh River for over 40 years.

### 2.5 Inference and model diagnostics

We estimated the integrated MARSS model on a joint Bayesian posterior in the probabilistic programming language Stan with 6 Markov Chain Monte Carlo (MCMC) chains using the “rstan” package in R (Carpenter et al., 2017; Stan Development Team, 2020). Each chain took 10,000 posterior samples with a warmup period of 50% for a total of 60,000 samples. Parameter values for each of the δ regression coefficients started at 0 for each chain. We used several complementary methods to diagnose model suitability. MCMC chain convergence was inspected visually on trace plots. In addition, we ensured effective sample sizes were >1,000 for each parameter (Gelman et al., 2013). We used the Gelman‐Rubin diagnostic test on each parameter to determine whether independent chains converged to a common posterior mode, with potential scale reduction factors (PSRF) <1.1 suggesting convergence. We then used graphical posterior predictive checks to test for model misspecification by comparing the predictive distribution of survival, adult returns, spawning time, and recruitment productivity (simulated from the posterior sample for each observation) to observed population dynamics. All environmental covariates (i.e., all $X_{t}$ in the above equations) were standardized to have zero‐mean and unit‐variance to directly compare effect sizes. Last, we inferred the weight of evidence for an association between environmental drivers on population dynamics from calculating the posterior probability that estimated coefficients were greater than (or less than) zero. Covariates with posterior probabilities closer to 100% indicate stronger certainty for a negative (or positive) association on Keogh salmonid population dynamics.

## 3 RESULTS

### 3.1 Ecosystem regimes

Since 1976, many marine and freshwater environmental factors associated with the Keogh salmonid community experienced either slow trends or fast fluctuations (Figure S3). In the ocean, the biomass of North Pacific Salmon and the abundance of coastal seal populations slowly increased during the 1980s and reached an asymptote during the early 2000s. Additionally, indices for the North Pacific Decadal Oscillation describing ocean productivity began 10‐year high‐to‐low cycles in 1976 (the start of the Keogh time‐series). This has led to recent marine and coastal ecosystem regimes associated with higher marine competition (2.4‐fold increase in North Pacific salmon abundances), higher coastal predation risk (eightfold increase in seal densities), and cyclical ocean productivities. The freshwater environment in recent decades generally shifted into a period of higher clear‐cut logging with high returns of Pink Salmon in some years that provide resources to stream‐rearing salmonids and variable climates associated with warming trends and variation in when adult salmon returned to the river. Specifically, the cumulative logged area over a 15‐year integration period slowly increased throughout the 1980s before reaching an asymptote in the late 1990s and declining in the late 2000s. The climate of the inland environment has also varied annually with changing air temperatures and rainfall, which likely affect river temperatures and flow levels. During times for winter spawning salmonids, for example, some winters were warm and wet while others were cooler and drier.

### 3.2 Community dynamics

**FIGURE 3:**
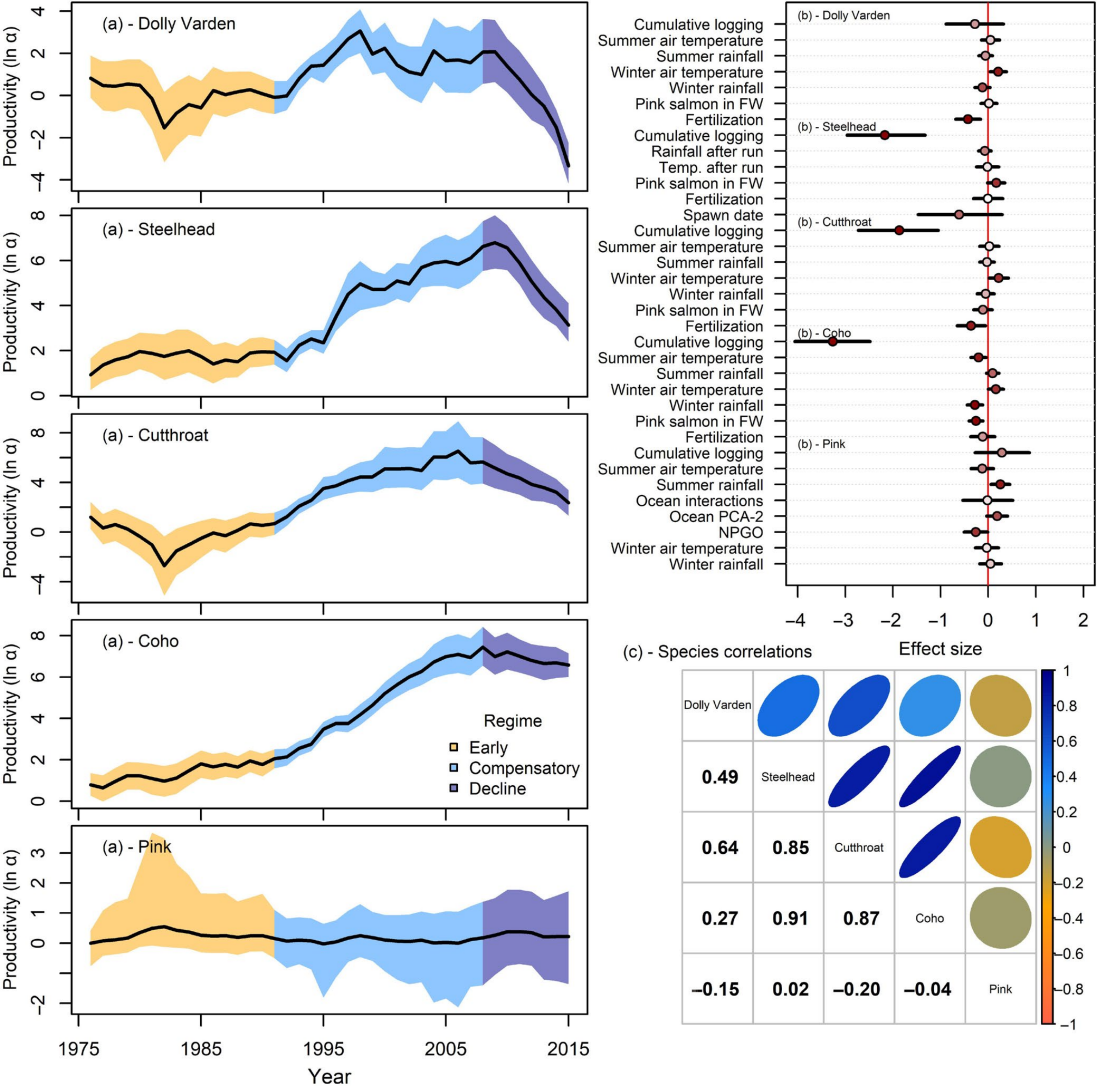
Posterior predictive distribution (mean and 80% credible intervals (CI) indicated by lines and shaded polygon, respectively) of trends in intrinsic productivity (a) and emergent ecological regimes (a – shaded regions) for five salmonids through time. Mean effect sizes (panel b – points) and 80% credible intervals (panel b – line) of environmental drivers on recruitment productivity. Point colors in panel b indicate relative strength of inference for each covariate—points closer to dark red indicate closer to 100% posterior probabilities that estimated coefficients were positive (or negative) and points closer to white indicate weak or no support. Posterior mean correlation between species’ intrinsic productivity through time (panels c)

Three freshwater productivity regimes appear to have emerged for the Keogh salmonid community over the past 40 years based on shared persistent trends in intrinsic productivity, but the strength of each regime varied between species (Figure 3a). First, we defined an “early regime,” where population dynamics were generally stable from 1976 to 1990 with low or moderate intrinsic productivity (i.e., ln(α) for all five species). Next, we defined a “compensatory regime,” where intrinsic productivity of four species (Dolly Varden, Steelhead, Coastal Cutthroat, and Coho) increased substantially from 1991 to 2007 (i.e., increased ln(α) in Equation 7), which manifested as a nonstationary shifting intercept in the stock–recruit relationship. However, we found evidence for a third “declining regime,” where intrinsic productivity appeared to lower for three species (Dolly Varden, Steelhead, and Coastal Cutthroat) despite adult numbers remaining low since ~2007 (Figure S4). For example, intrinsic productivity ln(α) of Steelhead increased by 109% (80% credible intervals [CI]: 50–439%) from 1976 to 1991 as the population declined but $\ln\left(\alpha \right)$ declined by 63% (80% CI: 65–74%) from 1991 to 2015. Estimated model parameters passed all diagnostic and MCMC convergence checks (e.g., all PSRFs <1.1) with low percent error, and the posterior predictive distribution for recruitment productivity indicated reasonable model fit to the observed time‐series (Figure S5).

Productivity regime shifts were associated with species’ life histories (Table 1; Figure 3a). Species with longer freshwater residence and overall lifespans experienced larger regime shifts, the greatest declines, and the most persistent population depression. Specifically, the species with extended freshwater residency—Dolly Varden, Cutthroat, and Steelhead—experienced the greatest changes in productivity, followed by Coho, which spend an intermediate amount of time in freshwater, and then Pink Salmon which migrate to sea as fry. Thus, it appears unlikely that these regimes shifts were exclusively associated with the marine environment. Interestingly, the productivity regime of Pink Salmon appeared stationary and annual variation in productivity was defined more by variance than by environmental change or systematic trends.

Species varied in the environmental factors associated with their observed recruitment productivity $\ln\left(\frac{R_{t}}{S_{t}}\right)$ (Figure 3b). Overall, all five species exhibited density dependence (100% of the posterior distribution for β < 0) and recruitment productivity increased as adult stocks decreased. In addition to density dependence, recruitment productivity for much of the community was negatively associated with the cumulative logging index. More than 99% of the posterior distribution for $\delta_{\mathrm{logging}}$ was below 0 for Steelhead, Cutthroat, and Coho, and 72% was below 0 for Dolly Varden. Interestingly, only 25% of the posterior distribution for $\delta_{\mathrm{logging}}$ was below 0 for Pink Salmon suggesting little association between Pink Salmon productivity and logging. Given that logging activities intensified over the 40 years, the predicted marginal effects of logging (all else equal) was a 97% decline in Steelhead smolts produced per adult female (80% CI: 88–99%), a 98% decline in Coho smolts produced per adult (80% CI: 95–99%), and a 99% decline in Cutthroat smolts produced per adult (80% CI: 91–99%) from 1976 to 2015. Climate factors had inconsistent associations with recruitment productivity (Figure 3b). For example, some climate changes were associated with increased recruitment productivity (Pink Salmon and summer rainfall; Coho Salmon and warmer winters; Dolly Varden and warmer winters). However, some climate signals were associated with decreased productivity (Coho and warmer summers; Dolly Varden and wetter winters; Pink Salmon and North Pacific Gyre Oscillation). The nutrient enrichment and fertilization experiments were negatively associated with recruitment productivity for Dolly Varden and Coastal Cutthroat but did not have substantial impacts on recruitment productivity of other species, even though past work has found that these experiments increased somatic growth of Steelhead (e.g., Bailey et al., 2018).

There was some evidence for species interactions and covariation within the community (Figure 3c). For example, the abundance of spawning Pink Salmon in Steelhead brood‐years was positively associated with higher observed recruitment productivity in (88% of the posterior distribution >0), suggesting that Pink Salmon may facilitate increased Steelhead productivity in their early life. In general, intrinsic productivity appeared synchronous for much of the community with intrinsic productivity going up and down together (pairwise correlations between Dolly Varden, Steelhead, Coastal Cutthroat, and Coho Salmon >0.5). As well, there were weak negative pairwise correlations between the intrinsic productivity of Pink Salmon and both Dolly Varden (−0.15) and Coastal Cutthroat (−0.2).

### 3.3 Steelhead life cycle

**FIGURE 4:**
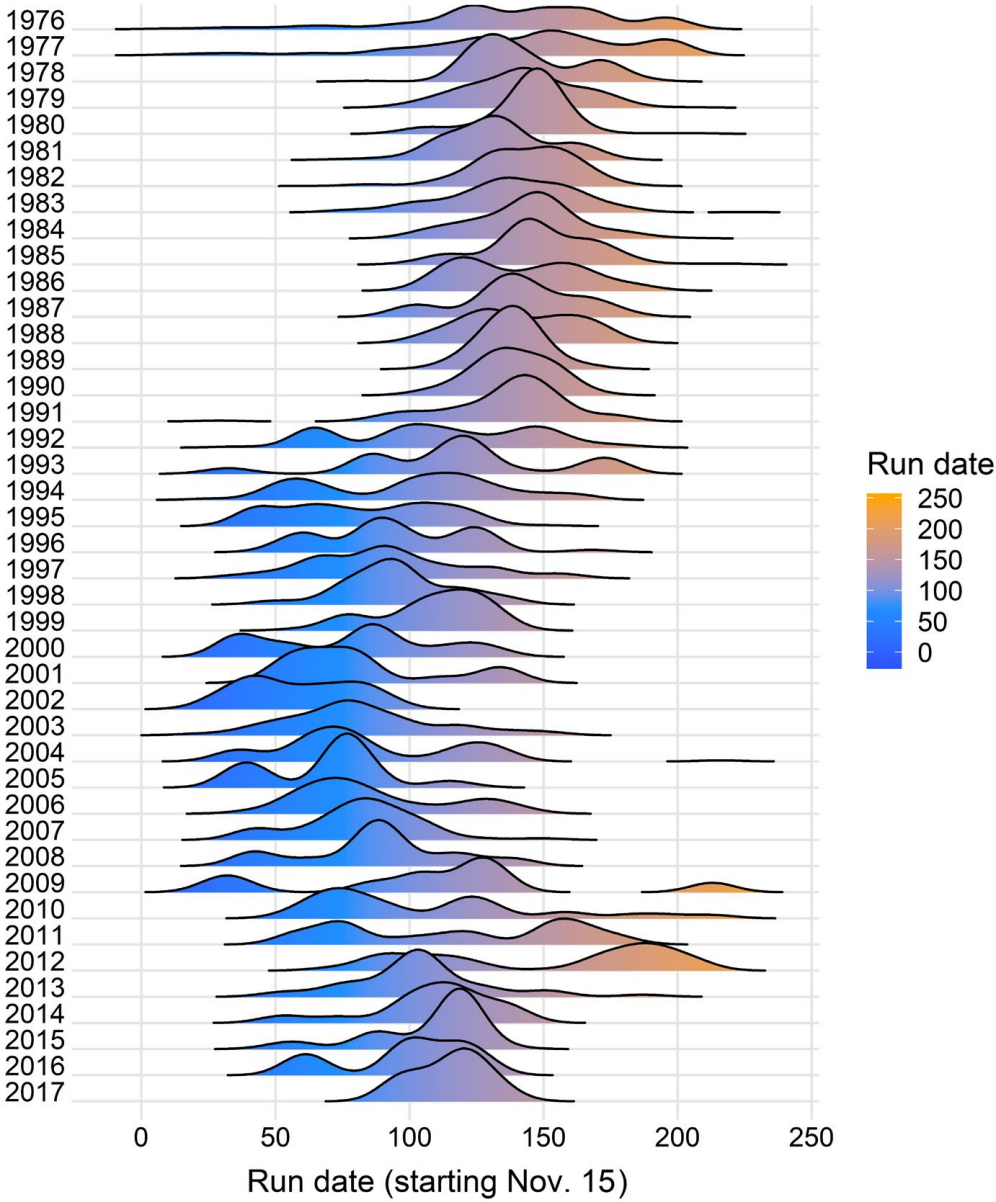
Distribution of spawning run dates for adult steelhead on the Keogh River since 1976

**FIGURE 5:**
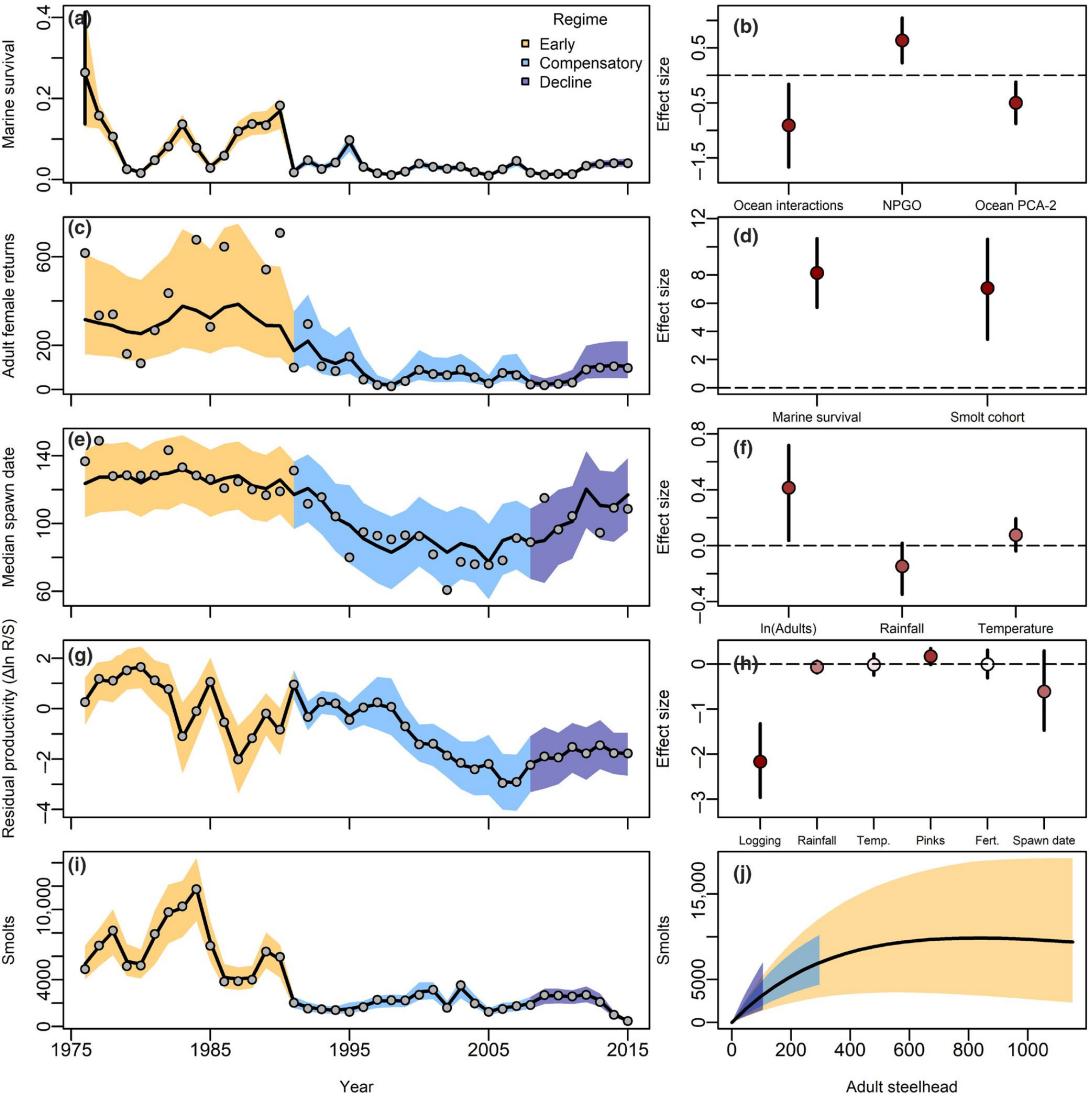
Observed (grey points) and posterior predictive distribution (mean and 80% credible intervals (CI) indicated by lines and shaded polygon, respectively) of ecological regimes across the steelhead life cycle. Marine survival trends (a) and environmental effects on marine survival (b). Adult returns (c) and environmental effects on adult returns (d). Spawning dates (e) and environmental effects on spawning dates (f). Residual productivity (g; the remaining smolt productivity not explained by time‐varying a and density dependence) and environmental effects on smolt productivity (h). Emergent recruitment dynamics (i) and recruitment bottlenecks compared with early regime (j). Point colors in sight‐side panels indicate relative strength of inference for each covariate—points closer to dark red indicate closer to 100% posterior probabilities that estimated coefficients were positive (or negative) and points closer to white indicate weak or no support

Marine and freshwater regime shifts were apparent across the Steelhead life cycle (Figures 4 and 5). Multiple changes in the marine and freshwater environment were associated with Steelhead being limited to low population sizes. For example, declining trends in marine survival since 1990 (Figure 5a) were associated with increased ocean species interactions (i.e., seal densities and North Pacific salmon abundance; Figure 5b) that may manifest as increased predation and competition. Lower adult returns were associated with earlier run times (Figures 4 and 5f). For example, upstream migration began ~32 days earlier in the “compensatory regime” than the “early regime,” and ~21 days earlier in the “declining regime” than the “early regime.” Subsequently, earlier run times were weakly associated with increased smolt productivity (79% of the posterior distribution was negative; Figure 5h). Although uncertain, this suggests that changing phenology may be linked with environmental stressors. Importantly, smolt cohort abundance and marine survival had approximately equal effect sizes on adult returns (Figure 5d), suggesting that the freshwater and marine portions of their life cycle had similar impacts on the overall dynamics of adult numbers.

Overall, the posterior predictive distribution of marine survival and observed recruitment productivity suggested that smolt production remains limited to low densities (Figure 5g and 5j). This bottleneck emerged despite the low spawner densities that should otherwise increase per‐capita productivity, assuming compensatory density dependence. Residual variation in steelhead smolt productivity (e.g., remaining variation not explained by density dependence) appeared to decline throughout the 1990s associated with large changes in freshwater covariates including forestry logging practices (Figure 5g). Since 2007, residual productivity has improved moderately, but total recruitment remains low. Given the average environment within each regime, the posterior predictive distribution suggests this recruitment bottleneck has been maintained by an interplay between density dependence, changing freshwater environments, and low marine survival.

## 4 DISCUSSION

We found evidence that regime shifts in the marine and freshwater environments structured the recruitment productivity regimes of a diverse anadromous salmonid community and that the strength of these shifts varied with species’ life histories. Animal movement and migrations (in this case of Pacific salmonids) can connect not only ecosystems but also the potential drivers that underly regime shifts. Our results further support the idea that environmental change can have hidden feedbacks that change or weaken the typical processes that regulate populations, like density dependence, leading to nonstationary productivity regimes (Litzow et al., 2018; Rocha et al., 2018; Szuwalski & Hollowed, 2016). Our findings also suggest there may be stages of regime shifts beyond the classic two state model, such as long‐term transient population dynamics, which generate tremendous uncertainty for resource managers aiming to avoid catastrophe (Francis et al., 2021). Specifically, Steelhead in our study displayed regimes of (1) high marine survival and low‐moderate freshwater productivity transitioned into a regime of (2) low marine survival, high freshwater productivity, and alarmingly into a regime of (3) low marine survival and low freshwater productivity.

Cascading regime shifts can emerge from multiple environmental changes that cross ecological scales (Folke et al., 2004; Rocha et al., 2018). In British Columbia, for example, persistent changes in logging practices, the recovery of harbour seals (*Phoca vitulina*) and Steller sea lions (*Eumetopias jubatus*)after federal protection in Canada and the United States in the 1970s, and marine climate oscillations appear to have systematically altered the coastal and freshwater ecosystems that shape salmonid communities (Malick et al., 2017; Mueter et al., 2002; Nelson et al., 2019; Tschaplinski & Pike, 2017; Walters, et al. 2020). Beginning in the 1990s, the Province of British Columbia increased allowances for clear‐cut logging to satisfy demands for timber and combat outbreaks of invasive pests (Peel, 1991), and the current rate of deforestation in the province (6200 ha·yr^−1^) has remained steady in recent decades (Bourgeois et al., 2018; Gilani & Innes, 2020). Total impacts from forestry on Vancouver Island, in particular, are likely conservative due to underreporting that would underestimate the total extent of deforestation and associated impacts on Keogh salmonids over the past 40 years (Shackelford et al., 2018). Deforestation can lead to considerable slope and soil erosion (Guthrie, 2002; Harding & Ford, 1993) and modification of flow regimes (Gronsdahl et al., 2019), and the resultant downstream sediment deposition can decrease suitable Pacific salmonid habitat and production (Reid et al., 2020; Tschaplinski & Pike, 2017). Hence, systematic changes in one environment (e.g., terrestrial forestry practices or coastal seal recoveries) may have unknowingly triggered cascading regime changes that took years to manifest. For example, harbor seals took 20 years to recover from decades of overharvest and hydrological and habitat alterations can lag behind intensive logging by 10–30 years (Nelson et al., 2019; Reid et al., 2020; Tschaplinski & Pike, 2017). Thus, the migratory life cycles of anadromous fishes expose them to stressors in different ecosystems, and these stressors might only emerge decades after the management decisions occurred.

There is often an implicit and prevailing narrative that marine ecosystem changes are the strongest factor limiting anadromous salmonids and some have even asserted that investments in freshwater habitat conservation and restoration are misguided (e.g., Welch et al., 2020). Our findings suggest that population dynamics of anadromous salmonids may be equally limited to low densities by worsening freshwater and marine factors (see Figure 5d). These results add to previous work suggesting that strong density dependence in freshwater habitats can continue to limit productivity even in severely depressed salmon populations (Achord et al., 2003; Walters et al., 2013). However, mechanisms and patterns underlying these bottlenecks can manifest differently among species with diverse life histories. For example, with their fast life cycle and lack of a freshwater rearing phase, most Pink Salmon populations throughout the Pacific Northwest have not shown strong temporal trends in productivity (Malick & Cox, 2016), while Steelhead and Chinook Salmon, with their longer lifespans and greater dependence on freshwater habitat, are in serious decline (Dorner et al., 2018; Kendall et al., 2017).

Many wild salmonids are in widespread decline across the Pacific Northwest (Kendall et al., 2017; Peterman & Dorner, 2012; Walters et al., 2019). The role of freshwater density dependence in the status of at‐risk Pacific salmonids remains uncertain as relatively few studies have jointly monitored freshwater and marine life stages (Scheuerell et al., 2020; Walters et al., 2013). Many salmon populations in Canada, including Thompson River and Chilcotin River Steelhead, have been recently recommended for Endangered or Threatened at‐risk status with worsening ocean conditions frequently listed as a key driver of their decline (COSEWIC, 2018, 2020). Interestingly, Keogh River Steelhead Trout exhibit strong coherence with both Thompson and Chilcotin populations (Figure S6; COSEWIC, 2018). We quantified relatively equal contributions of freshwater productivity and marine survival on Steelhead population dynamics suggesting that there may be multiple bottlenecks available for management to target (Atlas et al., 2015; Scheuerell et al., 2020). Furthermore, we quantified strong covariation between the productivity regimes of Steelhead and both Coastal Cutthroat and Dolly Varden, both of which often have understudied population dynamics across their range. Given the strong covariation between Keogh species and between Steelhead populations, it is possible that similar rain‐dominated coastal watersheds with declining Steelhead populations may also have hidden declines in Dolly Varden and Coastal Cutthroat populations. Although a full recovery of at‐risk Pacific salmonids will likely rely upon improvements to the marine environment that might have relatively limited local management levers, provincial‐scale management could aim to target freshwater bottlenecks, like the restoration of watershed hydrology that might have been altered through logging. Managing and restoring function to watersheds could help maintain Steelhead and other species in rain‐dominated watersheds, like the Keogh River, common across the Pacific Northwest (Jones et al., 2020).

The legacy of regime shifts can have lasting impacts on the structure and functions of ecosystems (Achord et al., 2003; Szuwalski & Hollowed, 2016). Contemporary alterations to the physical or biological processes that structure ecosystems spatially (e.g., habitat connectivity and migration) and temporally (e.g., climate cycles and organism generation times) may have persistent effects on future generations and species via direct mortality, reduced performance, and carry‐over effects (Achord et al., 2003; Dorner et al., 2018). Here, we revealed how a diverse salmonid community may experience productivity shifts structured not only by density dependence but also by multiple ecosystem changes across their complex life histories. Nonstationary changes in recruitment productivity may exacerbate ongoing density dependent bottlenecks in territorial species, such as Pacific salmonids, presenting new challenges for conservation and management (Atlas et al., 2015; Scheuerell et al., 2020). Importantly, we demonstrate that wrapping estimates of population productivity across marine and freshwater ecosystems may allow the underlying productivity regime of one ecosystem to mask the others. Partitioning how freshwater and marine factors jointly shape the population dynamics of Pacific salmonids can help to reveal the ecosystem changes limiting their resilience, allowing pragmatic management to better target relevant bottlenecks.

## CONFLICT OF INTEREST

The authors declare that there is no conflict of interest.

## AUTHORS’ CONTRIBUTIONS

KLW led the project and conducted the analyses. KLW and JWM conceived the ideas for the manuscript. All authors wrote the manuscript and gave final approval for publication.

## Supporting information

Fig S1‐S6

## Data Availability

All data, R code, and the Stan model used for this manuscript are archived at https://doi.org/10.5281/zenodo.4977300.
