# Supplementary material for: Marine and freshwater regime changes impact a community of migratory Pacific salmonids in decline
Source: Glob Chang Biol. 2021 Oct 20;28(1):72–85. doi: 10.1111/gcb.15895 (PMC9298309; doi:10.1111/gcb.15895)
Supplement: Supplementary file 1 — Fig S1‐S6 [file GCB-28-72-s001.docx]

List of supplemental files in support of the main manuscript by Wilson et al. “Marine and freshwater regime changes impact a community of migratory Pacific salmonids in decline” in consideration at *Global Change Biology*.


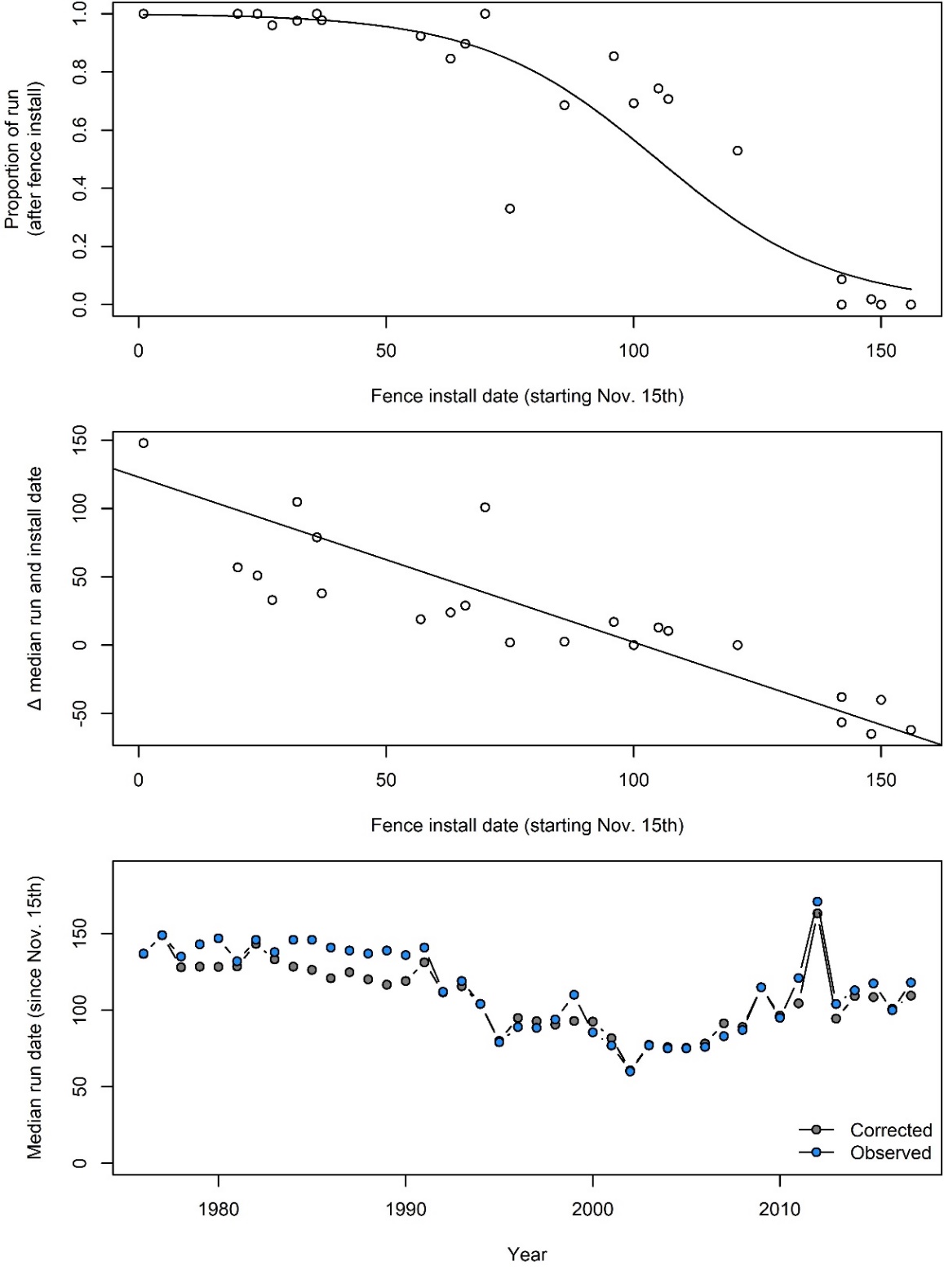


Figure S1 – Median spawning run date for adult Steelhead Trout corrected based on (a) the portion of the adult run available for sampling by the date of the fence installation for upstream monitoring on the Keogh River and (b) the mean difference between the median run date and the fence install date. Data used to make corrections were after 1997 when the resistivity counter was installed.


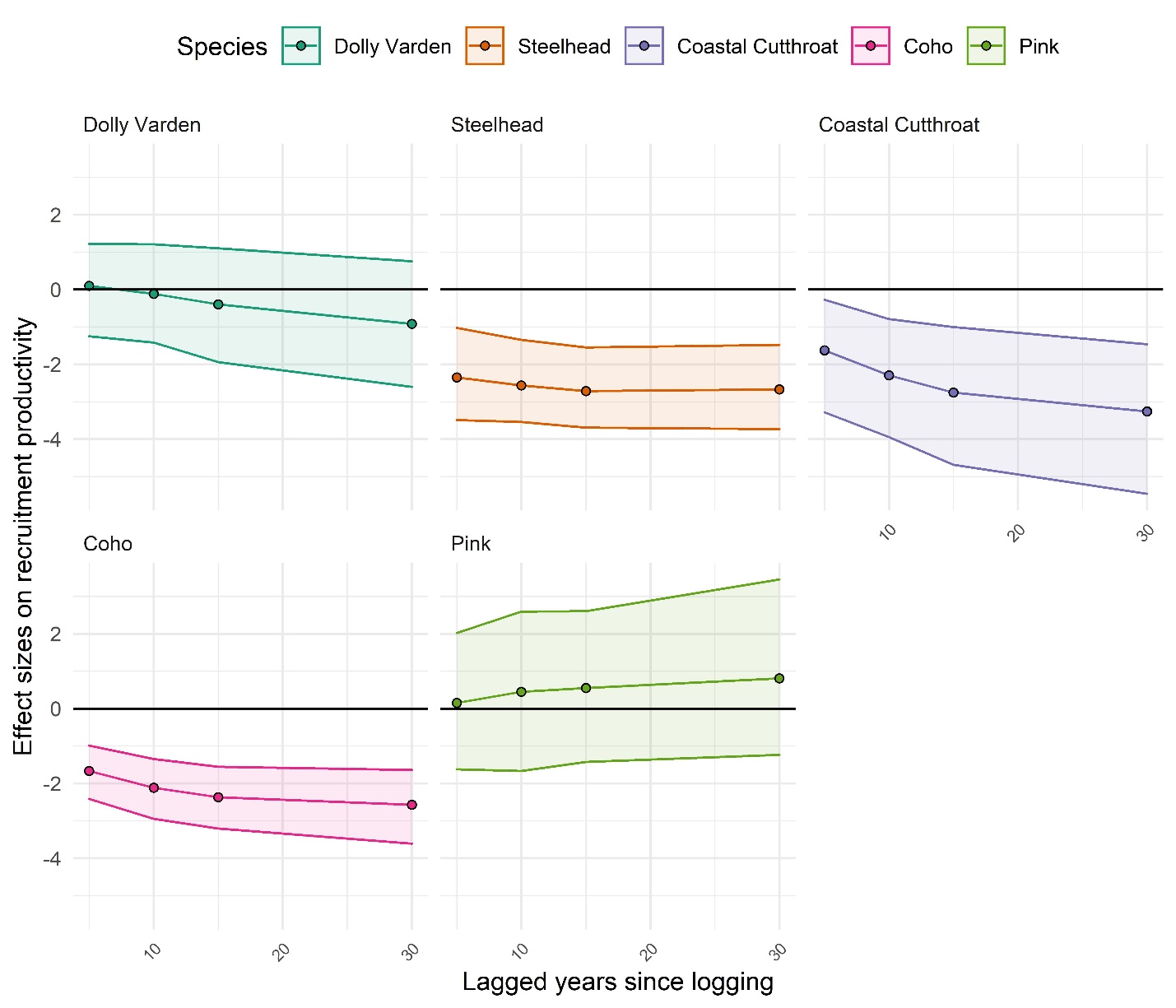


Figure S2 – Sensitivity test on the effect sizes along gradients of alternate time lags on forestry logging impacts to Keogh salmonid recruitment productivity. A 15-year time lag was used in the main paper. Points indicate posterior mean effect sizes in points, and shaded polygon indicates 95% credible intervals.


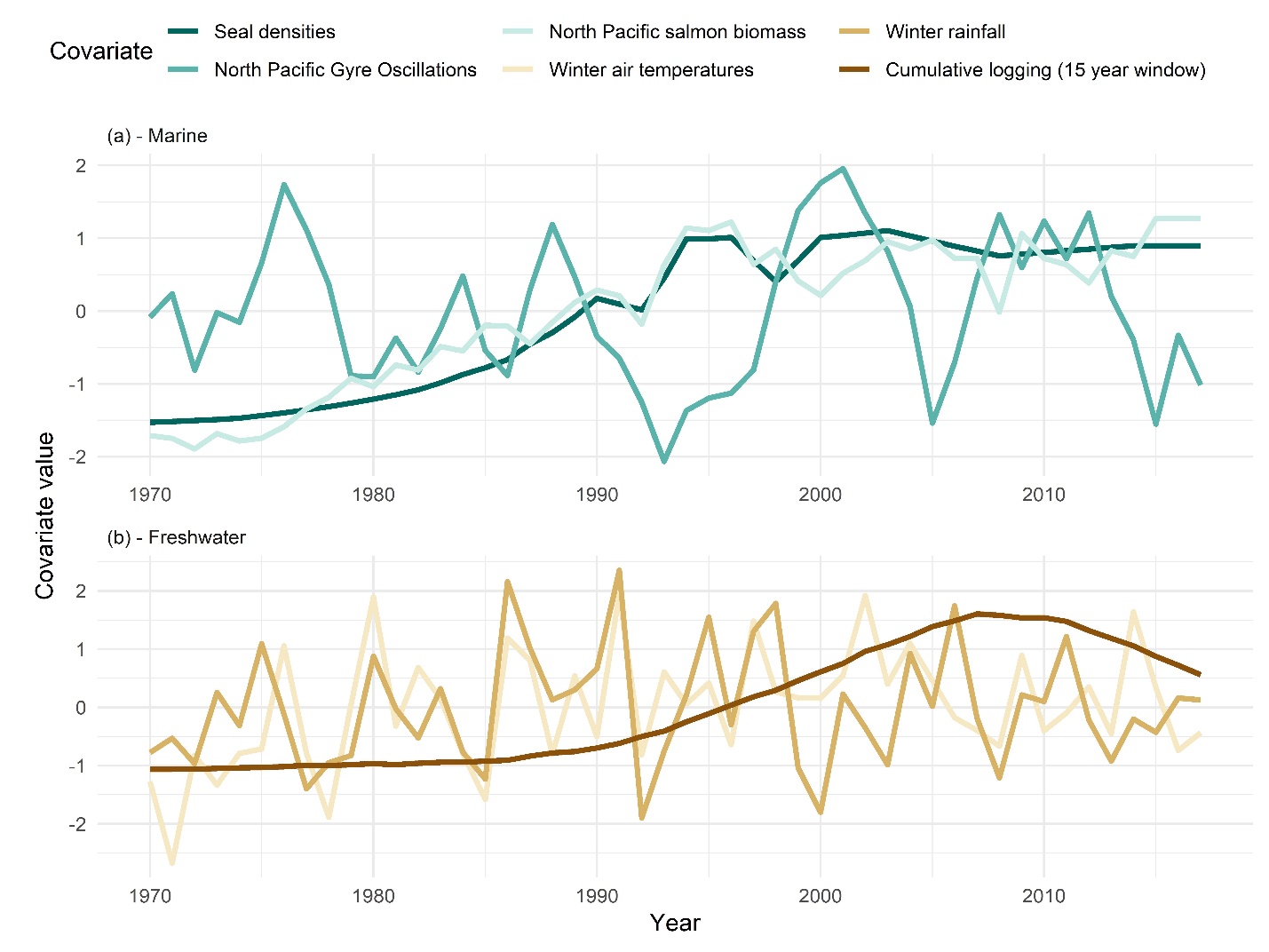


Figure S3 – Changing marine and freshwater environments in the Keogh River.


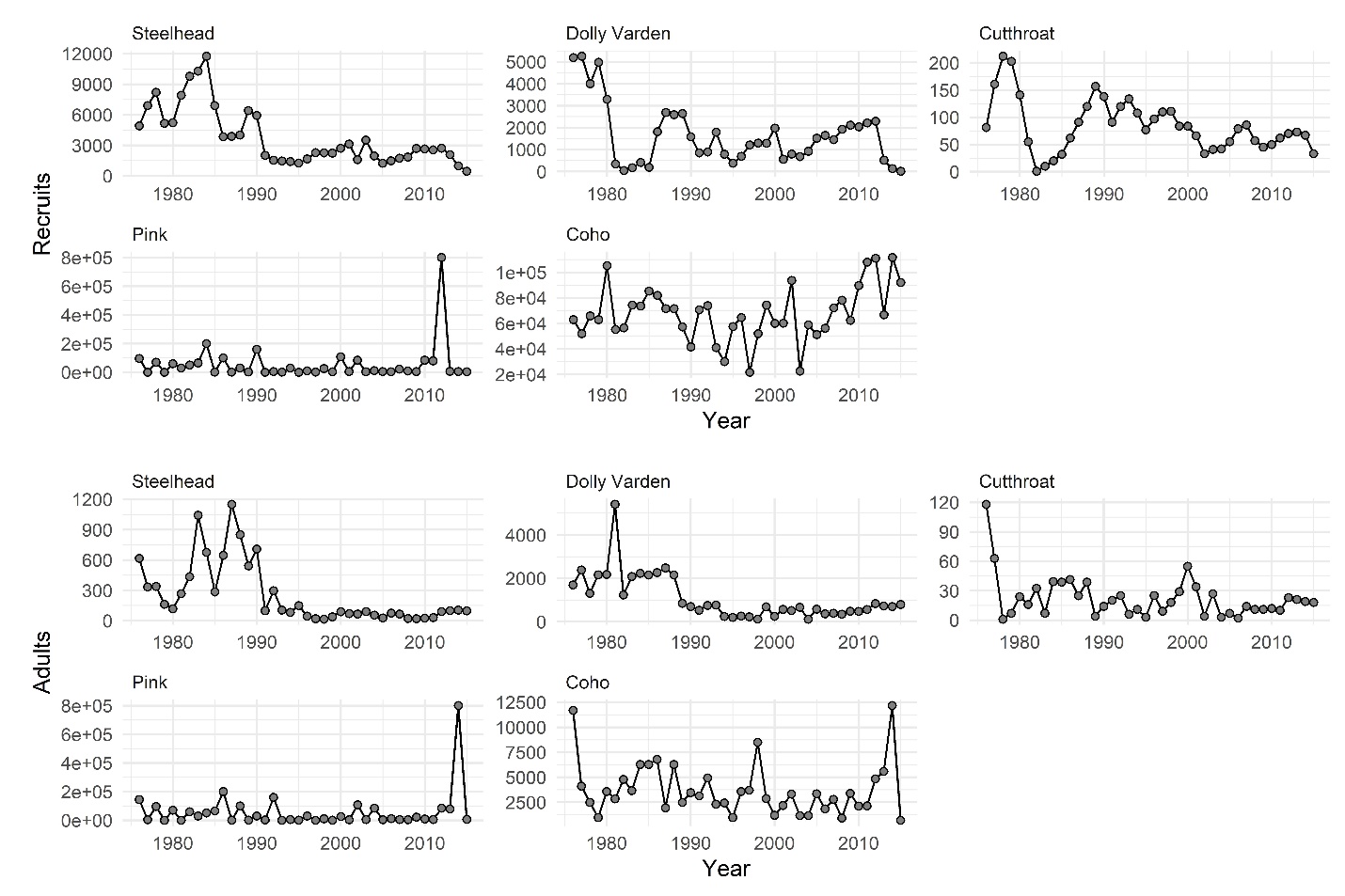


Figure S4 – Time series of recruits and spawners for five species of Pacific salmonids since 1976 on the Keogh River.


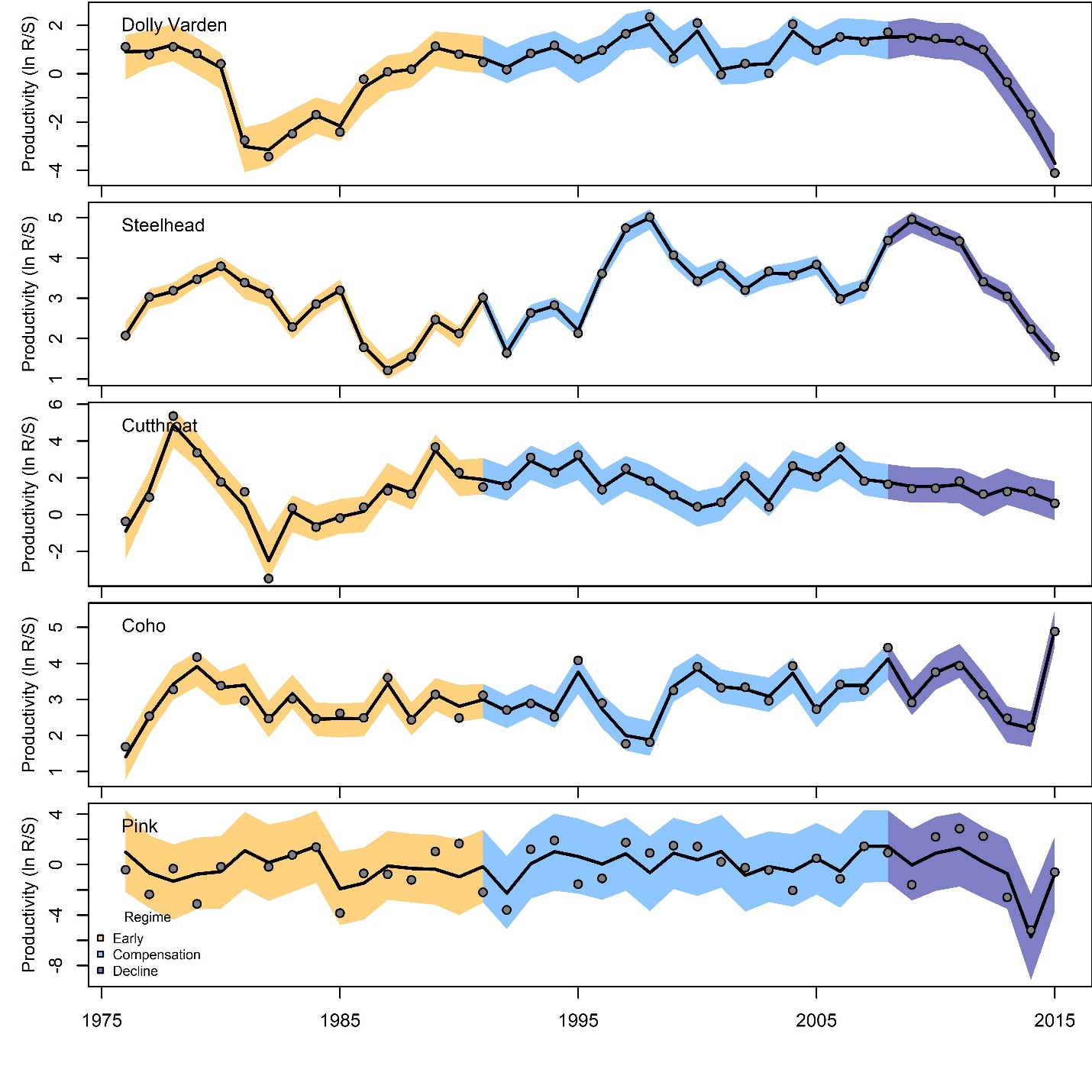


Figure S5 – Observed (points) and posterior predictive distribution (mean and 80% credible intervals (CI) indicated by lines and shaded polygons, respectively) for recruitment productivity of Pacific salmonids on the Keogh River since 1976.


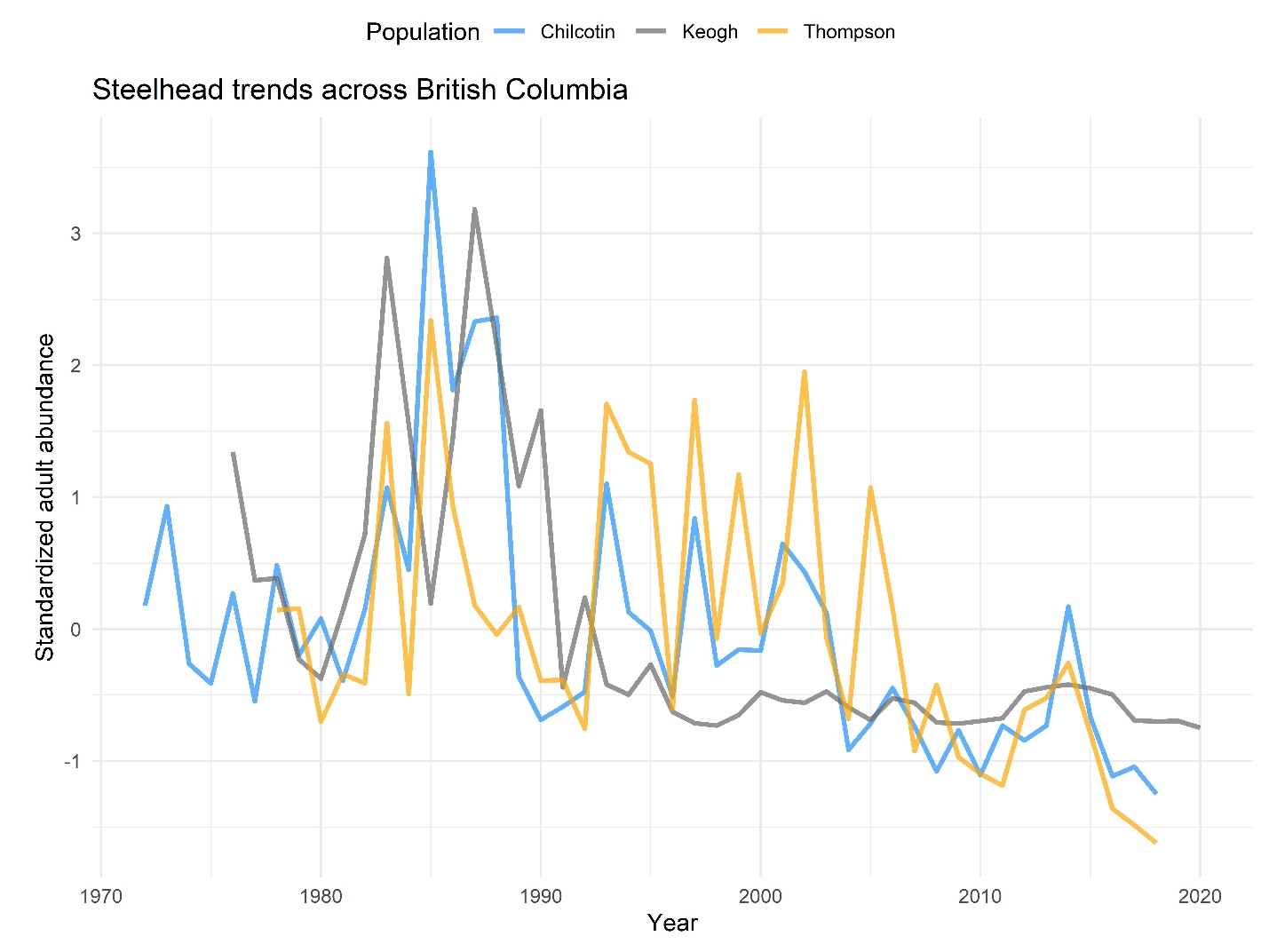


Figure S6 – Adult steelhead returns in the Chilcotin, Thompson, and Keogh Rivers through time. In 2018, the Committee on the Status of Endangered Wildlife in Canada performed an emergency assessment of both the Thompson and Chilcotin Steelhead Trout populations and found them to be endangered.
